# The Effect of TGF-β3 and IL-1β on L-Type Voltage-Operated Calcium Channels and Calcium Ion Homeostasis in Osteoarthritic Chondrocytes and Human Bone Marrow-Derived Mesenchymal Stem Cells During Chondrogenesis

**DOI:** 10.3390/pharmaceutics17030343

**Published:** 2025-03-07

**Authors:** Anastasiia Shelest, Aidas Alaburda, Raminta Vaiciuleviciute, Ilona Uzieliene, Paulina Bialaglovyte, Eiva Bernotiene

**Affiliations:** 1 Institute of Biosciences, Life Sciences Center, Vilnius University, LT-10257 Vilnius, Lithuania; 2 Department of Regenerative Medicine, State Research Institute Centre for Innovative Medicine, LT-08406 Vilnius, Lithuania; raminta.vaiciuleviciute@imcentras.lt (R.V.); ilona.uzieliene@imcentras.lt (I.U.); paulinabialaglovyte@gmail.com (P.B.); eiva.bernotiene@imcentras.lt (E.B.); 3 VilniusTech Faculty of Fundamental Sciences, Department of Chemistry and Bioengineering, Vilnius Gediminas Technical University, LT-10223 Vilnius, Lithuania

**Keywords:** TGF-β, IL-1β, intracellular calcium levels, voltage-operated calcium channels, L-type calcium channels, bone marrow mesenchymal stem cells, chondrocytes, chondrogenesis

## Abstract

**Background:** Transforming growth factor-β (TGF-β) and interleukin 1β (IL-1β) are key regulators of the chondrogenic differentiation, physiology and pathology of cartilage tissue, with TGF-β promoting chondrogenesis and matrix formation, while IL-1β exerts catabolic effects, inhibiting chondrogenesis and contributing to cartilage degradation. Both cytokines alter the intracellular calcium ion (iCa^2+^) levels; however, the exact pathways are not known. **Objectives:** This study aimed to evaluate the impact of TGF-β3 and IL-1β on calcium homeostasis in human bone marrow-derived mesenchymal stem cells (hBM-MSCs) and chondrocytes during chondrogenesis. **Results:** TGF-β3 increased iCa^2+^ levels in both hBM-MSCs and chondrocytes. Furthermore, TGF-β3 increased the functional activity of L-type voltage-operated calcium channels (L-VOCCs) in hBM-MSCs but not in chondrocytes. TGF-β3 and IL-1β reduced L-VOCCs subunit CaV1.2 (*CACNA1C*) gene expression in chondrocytes. In hBM-MSCs, TGF-β3 and IL-1β increased SERCA pump (*ATP2A2*) gene expression, while in chondrocytes, this effect was observed only with TGF-β3. **Conclusions:** TGF-β3 increases iCa^2+^ both in osteoarthritic chondrocytes and hBM-MSCs during chondrogenesis. In hBM-MSCs, TGF-β3-mediated elevation in iCa^2+^ is related to the increased functional activity of L-VOCCs. IL-1β does not change iCa^2+^ in osteoarthritic chondrocytes and hBM-MSCs; however, it initiates the mechanisms leading to further downregulation of iCa^2+^ in both types of cells. The differential and cell-specific roles of TGF-β3 and IL-1β in the calcium homeostasis of osteoarthritic chondrocytes and hBM-MSCs during chondrogenesis may provide a new insight into future strategies for cartilage repair and osteoarthritis treatment.

## 1. Introduction

Molecular mechanisms that control stem cell chondrogenic differentiation, chondrocyte homeostasis and cartilage extracellular matrix (ECM) formation have been of great interest for the past few decades. Transforming growth factor-β (TGF-β) is a cytokine and among the most important growth factors in the early stage of chondrogenesis, as well as the physiology and pathology of cartilage tissue [1]. Currently, the TGF-β family of proteins is used to induce chondrogenic differentiation in various source mesenchymal stem cells (MSCs), including those that are adipose-derived (AD-MSCs) [2], menstrual blood-derived (MenMSCs) [3] and bone marrow-derived (hBM-MSCs) [3,4,5,6,7,8]. In contrast, interleukin 1β (IL-1β) is a cytokine and a key mediator of the inflammatory response that is associated with the activation of catabolic pathways in cartilage and chondrogenesis inhibition, leading the cartilage tissue to degradation and osteoarthritis (OA) development [9,10,11,12].

TGF-β and IL-1β signal through various overlapping pathways including Ca^2+^ signaling, Smad1/5/8 and Smad2/3 pathways. It is known that low TGF-β levels stimulate Smad2/3 signaling, maintaining the chondrogenic phenotype [13,14], while IL-1β or high levels of TGF-β activate Smad1/5/8 signaling, leading to chondrocyte hypertrophy [13,15,16].

Elevated intracellular calcium ion (iCa^2+^) levels have been demonstrated to improve the chondrogenic differentiation of chicken MSCs [17]. On the other hand, elevated iCa^2+^ can activate Ca^2+^/calmodulin-dependent protein kinase II (CaMKII) associated with chondrogenic hypertrophy [18,19].

Regulation of iCa^2+^ plays a pivotal role in both hBM-MSC chondrogenesis and chondrocyte homeostasis, thereby influencing the intracellular pathways essential for cartilage formation and maintenance and regulating key processes such as proliferation, differentiation, and ECM production [20,21,22].

TGF-β induces Ca^2+^ influx in murine fibroblasts, mesangial cells, insulinoma cells, and human pulmonary fibroblasts [23,24,25,26]. IL-1β also mediates the elevation of iCa^2+^ in bovine chondrocytes [27].

It was observed that TGF-β led to a significant elevation in iCa^2+^ in rat chondrocytes via a few types of voltage-operated Ca^2+^ channels [28], while IL-1β was shown to mediate iCa^2+^ elevation in rat chondrocytes via the transient receptor potential ankyrin 1 (TRPA1) cation channel [29]. However, the mechanism of TGF-β- and IL-1β-induced changes in iCa^2+^ in human chondrocytes and hBM-MSCs remains unclear.

Potential mediators for iCa^2+^ regulation in this type of cell are L-type voltage-operated calcium channels (L-VOCCs). These channels are expressed in chondrocytes [30,31,32] and are sensitive to mechanical load [33,34,35]. The α1C subunit of L-VOCCs is highly expressed in hBM-MSCs [32,36]. However, the presence of the α1C subunit does not always result in functionally active channels. Only about 15% of undifferentiated hBM-MSCs demonstrated a small dihydropyridine-sensitive calcium current, mediated by L-VOCCs, under high external calcium concentration [37], indicating a low frequency of functionally active channels [36]. This may occur because the channels can be in an inactive state.

Despite all of the studies carried out before, it is still unclear what the mechanism is behind both the anabolic (TGF-β3) and catabolic (IL-1β) protein influence on iCa^2+^ concentration and how it affects chondrocyte homeostasis during the development of cartilage, and/or during the onset of OA. Therefore, the aim of this study is to investigate how TGF-β3 and IL-1β affect Ca^2+^ homeostasis in hBM-MSCs and chondrocytes during chondrogenesis.

## 2. Materials and Methods

### 2.1. Cell Isolation and Culture

Human tissue samples were obtained in accordance with the Bioethics Committee, permission No. 158200-14-741-257, from Vilnius University Hospital Santaros Klinikos. Articular cartilage samples were obtained as tissues removed during articular surgery from 4 patients with OA (aged 64 ± 14 years) without systemic, acute or chronic comorbidities. Chondrocytes were isolated and cultured according to the established protocols as previously reported [38]. Briefly, cartilage samples were washed in PBS with 1% penicillin-streptomycin (PS) (Gibco, Life Technologies, Waltham, MA, USA), chopped into small pieces, incubated in low glucose (1 g/L) Dulbecco’s Modified Eagle media (DMEM) (Capricorn Scientific, Ebsdorfergrund, Germany) with 1% PS at 37 °C in 5% CO_2_. After, samples were washed and enzymatically digested: first with pronase for 1 h, then with a type II collagenase solution at 10 mL/g of tissue for 4 h, both at 37 °C in 5% CO_2_. Isolated chondrocytes were cultured in complete medium, consisting of low glucose DMEM, 1% PS and 10% fetal bovine serum (FBS), in a 37 °C incubator with 5% CO_2_, changing the medium twice a week.

hBM-MSCs were isolated according to the established protocols by the Innovative Medicine Center (IMC) from 3 donors with OA (aged 52 ± 10 years), remaining after joint replacement surgical procedures. hBM-MSCs were incubated under the same conditions as chondrocytes, with the addition of 1 ng/mL of fibroblast growth factor 2 (FGF2) (Sigma Aldrich, Burlington, MA, USA) to maintain their stem cell potential and to avoid spontaneous differentiation. Isolated hBM-MSCs were cultured with complete medium in a 37 °C incubator with 5% CO_2_, changing the medium twice a week.

Passage 2–3 (P2–P3) of hBM-MSCs and chondrocytes were used for all experiments.

### 2.2. Chondrogenic Differentiation

Proliferation media consisted of DMEM media (with 1 g/L glucose), 10% FBS and 1% PS. Chondrogenic differentiation media was applied to hBM-MSCs and chondrocytes, consisting of high glucose (4.5 g/L) DMEM media, 1% PS, 1% insulin-transferrin-selenium (Gibco, Life Technologies, Waltham, MA, USA), 350 nM L-proline (Carl Roth, Karlsruhe, Germany), 100 nM dexamethasone (Sigma Aldrich, Burlington, MA, USA) and 170 nM ascorbic acid–phosphate (Sigma Aldrich, Burlington, MA, USA).

Chondrocytes and hBM-MSCs incubated in chondrogenic media were treated for 24 h with 10 ng/mL of TGF-β3 (Gibco, Life Technologies, Waltham, MA, USA) or with 10 ng/mL of IL-1β (Prospec, Ness-Ziona, Israel).

### 2.3. Intracellular Calcium Levels

The evaluation of iCa^2+^ levels was performed by seeding hBM-MSCs and chondrocytes in 6-well plates 200,000 cells/well. After the cells reached 95% confluence, subsequent incubation with IL-1β or TGF-β3 followed for 24 h, hBM-MSCs and chondrocytes were detached with 0.25% trypsin-EDTA, counted, and transferred at a density of 100,000 cells/vial into new 1.5 mL vials. These cells were then stained with Cal-520 dye (1 µM) (Interchim, Montlucon, France) for 30 min, washed with PBS, and measured using the Luminex Guava Flow cytometer (Luminex Corporation, Austin, TX, USA). The data were analyzed using FlowJo software, version 10 (FlowJo Corp., Ashland, OR, USA). 

### 2.4. Gene Expression Analysis

hBM-MSCs and chondrocytes were seeded in 6-well plates using 200,000 cell/well density. When cells reached 95% confluence, they were treated with IL-1β or TGF-β3 for 24 h. After, the cells were lysed using LTR lysis buffer (Qiagen, 74104, Hilden, Germany) and RNA was extracted according to the manufacturer’s instructions. The RNA concentration and purity of all samples were measured with SpectraMax i3 (Molecular Devices, San Jose, CA, USA). RNA was reverse-transcribed with a Maxima cDNA synthesis kit including dsDNase treatment (Thermo Fisher Scientific, Waltham, MA, USA). RT-qPCR reaction mixes were prepared with Maxima Probe qPCR Master Mix (Thermo Fisher Scientific, Waltham, MA, USA) and TaqMan Gene expression Assays (*RPS9*—Hs02339424_g1, *B2M*—Hs00984230_m1, *CACNA1C*—Hs00167681_m1, *ATP2A2*—Hs00544877_m1 (Thermo Fisher Scientific, Waltham, MA, USA)), and ran on the Agilent Aria MX instrument (Agilent Technologies, Santa Clara, CA, USA)) in technical triplicates starting with a denaturation step at 95 °C for 10 min followed by 40 cycles at 95 °C for 15 s of denaturation and 60 s for annealing and extension.

Relative levels of gene transcripts were calculated by subtracting the threshold cycle (Ct) of the normalizer (the geometric mean of the two housekeeping genes *RPS9* and *B2M*) from the Ct of the gene of interest, giving the dCt values that were subsequently transformed to 2-dCt values and multiplied by 1000 to scale-up for better graphical representation.

### 2.5. Electrophysiological Recording

For the electrophysiological recordings, hBM-MSCs and chondrocytes were seeded at 10,000 cells per coverslip and transferred to the recording chamber of a Nikon FN-S2N microscope (Nikon, Corporation, Tokyo, Japan). Membrane currents were measured in the whole-cell configuration of the patch-clamp technique at room temperature (21 ± 1 °C) with a Multiclamp 700B amplifier (Molecular Devices, San Jose, CA, USA) under the control of Clampfit 10.5 (Molecular Devices, San Jose, CA, USA). Patch microelectrodes were pulled from borosilicate glass capillaries with a Flaming/Brown micropipette puller (Model P-1000; Sutter Instrument Co., Novato, CA, USA). For whole-cell recordings, microelectrodes were filled with internal solution consisting of (mM): KCl 130, Na aspartate 10, MgATP 3, CaCl_2_ 0.2, EGTA 2, HEPES 5, (pH adjusted to 7.3 with KOH) [36,39]. The microelectrode tips’ resistance was ∼6–8 MΩ, when filled with internal solution. The presence of L-type calcium current (*I*_Ca,L_) was investigated with Ca^2+^ free Krebs–Ringer external solution (mM): NaCl 150, KCl 5.4, BaCl_2_ 10, MgCl_2_ 2, glucose 11, HEPES 10 (pH 7.4 adjusted with NaOH), where Ca^2+^ was substituted with Ba^2+^. This substitution increases the current flow through L-VOCCs, making it easier for identification [36,40].

Nifedipine (Sigma Aldrich, Burlington, MA, USA) and Bay-K8644 (Sigma Aldrich, Burlington, MA, USA) were used as blocker and activator, respectively. Both compounds were added to the external solution at a concentration of 10 µM before the current measurements.

Membrane currents were recorded using the voltage-clamp protocol that starts from a holding potential of −40 mV and a series of depolarizing steps applied in 10 mV increments to a maximum of +10 mV [41]. For isolation of the *I*_Ca,L_, we established I–V relationships for each analyzed cell using five data points of steady current of −30, −20, −10, 0, 10 mV, obtained from experimental data within 30 ms before the end of the depolarizing steps. Given that the observed peak influx of Ca^2+^ ions through L-VOCCs is noted at membrane potentials of 0 and +10 mV [36,42], we used three data points (−30, −20, −10 mV) to evaluate the leak conductance. The leak current at 0 and 10 mV was linearly extrapolated and then subtracted from experimental data, allowing the identification of the voltage-dependent component of the total membrane current, namely, the leak-subtracted current. The leak-subtracted and normalized to the leak current (*I*_LSN_) at +10 mV was used to evaluate the functional activity of L-VOCCs in both hBM-MSCs and chondrocytes. Negative *I*_LSN_ indicates the presence of inward voltage-operated current at +10 mV, while positive *I*_LSN_—outward voltage-operated current at +10 mV.

Our approach for determining the leak conductance involved using three data points (−30, −20, −10 mV) where a minor *I*_Ca,L_ was already present [36,42]. This implies that obtained values of *I*_LSN_ at +10 mV may be under-evaluated.

### 2.6. Statistical Analysis

The statistical difference between groups was evaluated using one-way analysis of variance (ANOVA) with OriginPro software, version 9.5 (OriginLab Corporation, Northampton, MA, USA), and a Student’s unpaired two-tailed *t*-test (Microsoft Excel) was used to calculate statistical significance. Significance was accepted when *p* ≤ 0.05. In the text, data are presented as mean ± standard deviation.

## 3. Results

### 3.1. Modulation of Intracellular Calcium Ion Levels in Response to TGF-β3 and IL-1β

**Figure 1:**
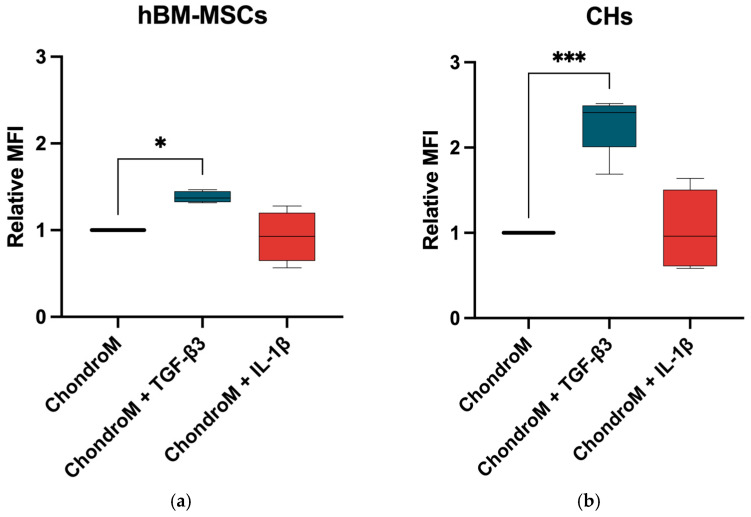
iCa^2+^ levels in (**a**) hBM-MSCs and (**b**) chondrocytes (CHs), after staining with Cal-520. The cells were cultivated in chondrogenic media (ChondroM) with IL-1β (10 ng/mL) and TGF-β3 (10 ng/mL) for 24 h. Median fluorescence intensity (MFI) is presented as a ratio to non-stained control. Measured with Luminex Guava. Data are presented as mean ± standard deviation of three technical repeats from no fewer than three OA patient’s cells. * *p* < 0.05, *** *p* < 0.001.

In order to determine how TGF-β3 and IL-1β can affect iCa^2+^ homeostasis in hBM-MSCs and chondrocytes, we examined their effects on iCa^2+^. TGF-β3 significantly increased iCa^2+^ in both hBM-MSCs and chondrocytes, while IL-1β did not show significant changes, as compared to chondrogenic media alone (Figure 1a,b).

### 3.2. Inward L-Type Calcium Current in hBM-MSCs

TGF-β3-mediated increase in iCa^2+^ depends on Ca^2+^ entry through L-VOCCs in insulinoma cells [25]. It is known that only 15% of undifferentiated hBM-MSCs demonstrated a small L-type calcium current (*I*_Ca,L_) [36,37]. However, the functional role of L-VOCCs in extracellular Ca^2+^ entry in hBM-MSCs is currently unknown. To investigate how TGF-β3 can cause elevation of iCa^2+^ in hBM-MSCs, we examined its impact on the functional activity of L-VOCCs in chondrogenic media. Additionally, the influence of IL-1β on L-VOCCs functional activity in hBM-MSCs was tested.

Chondrogenic media is enriched with high glucose DMEM, 1% PS, insulin-transferrin-selenium, L-proline, dexamethasone, and other bioactive compounds. Therefore, first we tested the effect of chondrogenic media alone on the functional activity of L-VOCCs.

**Figure 2:**
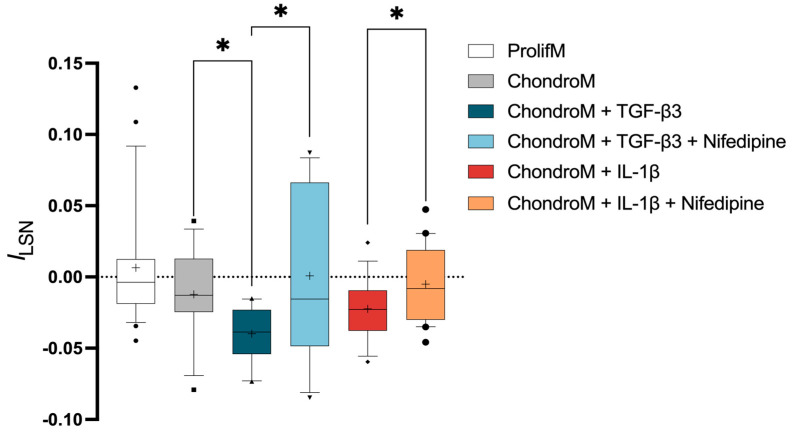
*I*_LSN_ in hBM-MSCs at +10 mV. The current was measured in cells cultivated in proliferation media (ProlifM) and chondrogenic media (ChondroM) with TGF-β3 (10 ng/mL) or IL-1β (10 ng/mL) for 24 h with or without nifedipine. Data, obtained from no fewer than three OA patient’s cells, are shown as a box with whiskers, indicating the median (50th), upper (90th), and lower (10th) percentiles. The number of cells for each condition are specified in the text. Outliers are represented with individual marks outside the whiskers. * *p* < 0.05.

hBM-MSCs cultivated in proliferation media did not show significant leak-subtracted normalized current at +10 mV (*I*_LSN_) (0.006 ± 0.04) in n = 22 cells (Figure 2). Cultivation in chondrogenic media did not show significant effects on *I*_LSN_ in hBM-MSCs (−0.01 ± 0.03; n = 12), compared to the proliferation media (Figure 2).

The exposure of hBM-MSCs to TGF-β3 caused significant negative *I*_LSN_ (−0.04 ± 0.02; n = 14), compared to chondrogenic media alone (Figure 2). TGF-β3-mediated negative *I*_LSN_ was sensitive to nifedipine, an L-VOCC blocker (0.0007 ± 0.06; n = 12) (Figure 2).

The exposure of hBM-MSCs to IL-1β showed a tendency to cause negative, but not statistically significant, *I*_LSN_ compared to chondrogenic media (−0.02 ± 0.02; n = 16) (Figure 2). Nifedipine resulted in significantly reduced *I*_LSN_ (−0.005 ± 0.03; n = 20) (Figure 2).

### 3.3. Inward L-Type Calcium Current in Chondrocytes

To investigate how TGF-β3 can cause the elevation of iCa^2+^ in chondrocytes, we examined its impact on the functional activity of L-VOCCs. Additionally, we investigated how IL-1β influenced L-VOCCs functional activity in chondrocytes.

**Figure 3:**
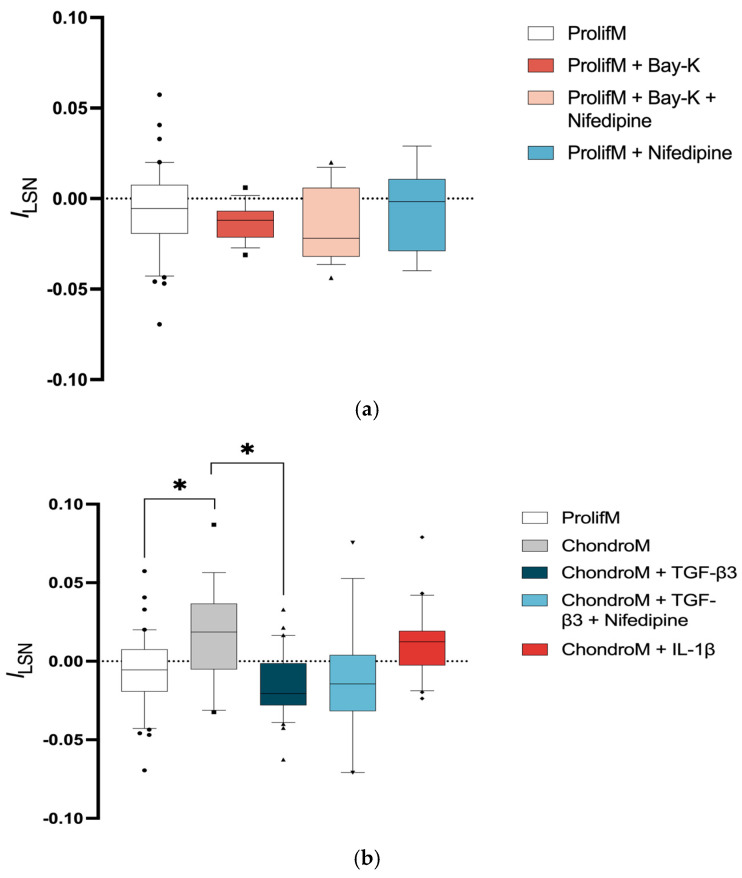
*I*_LSN_ in chondrocytes at +10 mV. The current was measured in cells cultivated (**a**) in proliferation media (ProlifM) with Bay-K8644, nifedipine, or both; (**b**) in proliferation and chondrogenic media (ChondroM) with TGF-β3 (10 ng/mL) or IL-1β (10 ng/mL) for 24 h with or without nifedipine. Data, obtained from no fewer than three OA patient’s cells, are shown as a box with whiskers, indicating the median (50th), upper (90th), and lower (10th) percentiles. The number of cells for each condition are specified in the text. Outliers are represented with individual marks outside the whiskers. * *p* < 0.05.

Chondrocytes cultivated in proliferation media did not show significant *I*_LSN_ (−0.007 ± 0.02; n = 42) (Figure 3a). The application of Bay-K8644, an activator of L-VOCCs, to proliferation media did not result in a significant change in *I*_LSN_ (−0.01 ± 0.01; n = 16) (Figure 3a).

The inward current could be compensated by voltage-operated potassium currents [43]. Moreover, the lack of changes in *I*_LSN_ after the application of Bay-K8644 may indicate that the majority of L-VOCCs were already activated. This hypothesis was tested by the application of nifedipine. Nonetheless, applying nifedipine or a combination of Bay-K8644 with nifedipine did not result in a significant change in *I*_LSN_ (−0.006 ± 0.02; n = 9, −0.01 ± 0.02; n = 18, respectively) (Figure 3a).

Incubation of chondrocytes in chondrogenic media caused significantly positive *I*_LSN_ (0.02 ± 0.03; n = 18), indicating the activation of voltage-operated outward current (Figure 3b).

The exposure of chondrocytes to TGF-β3 caused significantly negative *I*_LSN_ (−0.02 ± 0.01; n = 31) compared with chondrocytes cultivated in chondrogenic media alone (Figure 3b). TGF-β3-mediated negative *I*_LSN_ was not sensitive to nifedipine (−0.01 ± 0.04; n = 16) (Figure 3b).

The exposure of chondrocytes to IL-1β did not change *I*_LSN_ (0.01 ± 0.02; n = 20) when compared to chondrogenic media alone (Figure 3b).

### 3.4. Effect of TGF-β3 and IL-1β on CACNA1C and ATP2A2 Gene Expression

Both TGF-β3 [44] and increased iCa^2+^ levels [45,46] may contribute to homeostasis of Ca^2+^ at the gene expression level; therefore, we investigated how these two factors influenced the gene expression of Ca^2+^ regulators (L-VOCCs subunit CaV1.2, encoded by the *CACNA1C* gene and the SERCA pump, encoded by the *ATP2A2* gene) in hBM-MSCs and chondrocytes. Additionally, we analyzed IL-1β effects on the *CACNA1C* and *ATP2A2* gene expression.

**Figure 4:**
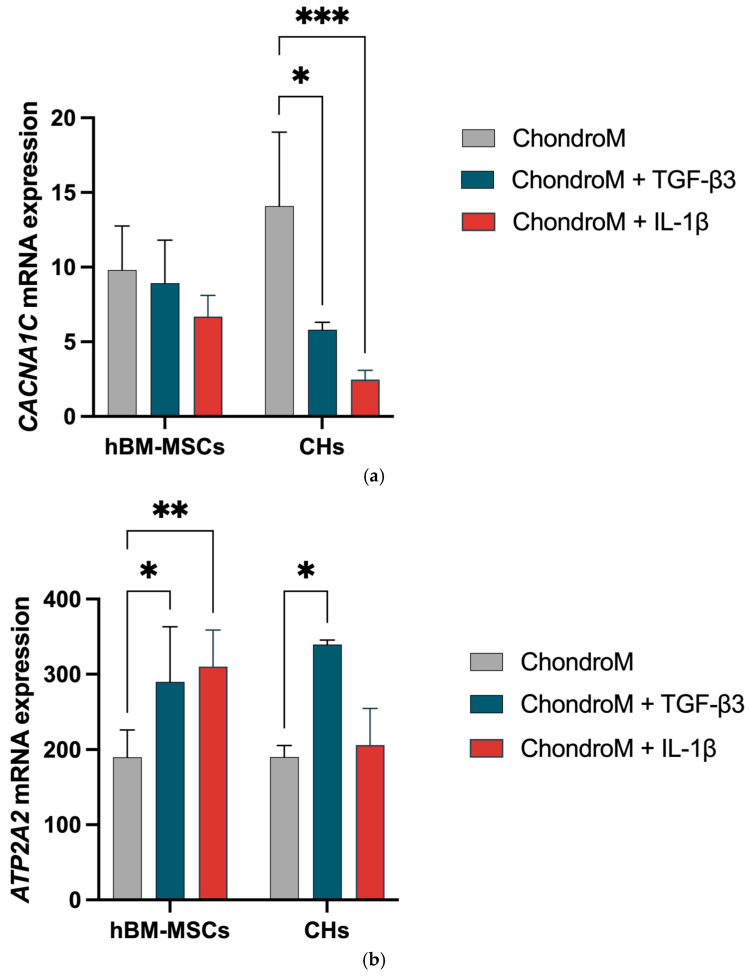
The effect of IL-1β (10 ng/mL) and TGF-β3 (10 ng/mL) on hBM-MSCs and chondrocyte (CHs) Cav1.2 (*CACNA1C*) and SERCA2 pump (*ATP2A2*) gene expression. The expression ratio of (**a**) *CACNA1C* and (**b**) *ATP2A2*, were analyzed in chondrogenic media (ChondroM) with (10 ng/mL) and IL-1β (10 ng/mL). Relative transcript level after normalization to the geometric mean of housekeeping *B2M* and *RPS9* genes are shown. Data are presented as mean ± standard deviation of three technical repeats from no fewer than three OA patient’s cells. * *p* < 0.05, ** *p* < 0.01, *** *p* < 0.001.

The *CACNA1C* gene expression was significantly downregulated in chondrocytes in response to both TGF-β3 and IL-1β; however, no effect was observed in hBM-MSCs (Figure 4a).

The *ATP2A2* gene expression was significantly increased in hBM-hMSCs in response to both TGF-β3 and IL-1β. In chondrocytes, the *ATP2A2* gene expression was significantly increased with exposure to TGF-β3 (Figure 4b).

## 4. Discussion

In this study, we investigated how 24 h exposure to 10 ng/mL of TGF-β3 and 10 ng/mL of IL-1β affect Ca^2+^ homeostasis in hBM-MSCs and chondrocytes during chondrogenesis.

Three aspects of Ca^2+^ homeostasis were evaluated: iCa^2+^ levels, *I*_Ca,L_, providing insights as to whether Ca^2+^ is entering cells through the L-VOCCs and, finally, expression of *CACNA1C* and *ATP2A2* genes which represent the cellular responses to the altered iCa^2+^ levels seeking to restore homeostasis.

We observed an increase in iCa^2+^ in both hBM-MSCs and chondrocytes following exposure to TGF-β3. The *I*_Ca,L_ in BM-hMSCs, but not in chondrocytes, was enhanced by TGF-β3. *CACNA1C* gene expression was significantly reduced in chondrocytes by both TGF-β3 and IL-1β, that also showed tendencies for a reduction in the *CACNA1C* gene in hBM-MSCs. The gene expression of *ATP2A2* was increased by TGF-β3 and IL-1β in hBM-MSCs, while in chondrocytes, just by TGF-β3.

The measure of 10 ng/mL of TGF-β3 was chosen for all experiments as it is a standard concentration used to induce chondrogenic differentiation [3]. Similarly, 10 ng/mL IL-1β was chosen based on the literature as it demonstrated early degenerative changes in human chondrocytes [47]. The 24 h incubation period was selected for all experiments to study early changes in cells.

There are three known isoforms of TGF-β expressed in mammals, including TGF-β1, TGF-β2, and TGF-β3. Their sequences are 71–80% identical, and they activate signaling through the same receptors [48]. Among its three isoforms, TGF-β1 or TGF-β3 are used for the induction of chondrogenesis [38,49,50]. We chose to use TGF-β3 because it was shown to have a higher potential to induce chondrogenic differentiation than TGF-β1 [51].

TGF-β3 mediated a significant elevation in iCa^2+^ in both hBM-MSCs and chondrocytes. Previous studies reported an elevation in iCa^2+^ as early as within 1 min after incubation with TGF-β1 in chondrocytes [28]. Moreover, an increase in iCa^2+^ mediated by TGF-β has been observed in other cell types, including neurons [52,53], osteoblasts [54], and fibroblasts [23,55].

IL-1β mediated an increase in iCa^2+^ in rat chondrocytes through TRPA1 [29]. Our research demonstrated that a 24 h exposure to 10 ng/mL IL-1β does not cause a change in iCa^2+^ in hBM-MSCs and chondrocytes, which corresponds to a previous study in this type of cells [38].

The regulation of iCa^2+^ in cells depends on Ca^2+^ entering through channels located on the plasma membrane, with L-VOCCs being one of them. In chondrocytes, L-VOCC activation is associated with the pathogenesis of osteoarthritis (OA), making them potential therapeutic targets for alleviating OA severity [22,31]. The increase in iCa^2+^ mediated by TGF-β3 may be related to changes in the functional activity of L-VOCCs. Therefore, we explored L-VOCCs’ functional activity by investigating *I*_LSN_ at +10 mV, which corresponds to the peak of the *I*_Ca,L_ [42]. The leak subtraction enabled the isolation of *I*_Ca,L_ from other (mainly potassium) currents present in the hBM-MSCs [36] and chondrocytes [56], while normalization minimized the effect of cell size. Incubation of hBM-MSCs with TGF-β3 resulted in an increased nifedipine-sensitive inward current, suggesting activation of L-VOCCs. This activation can result from a complex cascade reaction stimulated by TGF-β3. For example, it has been demonstrated that TGF-β increased β-adrenergic signaling [57]. The stimulation of β-adrenergic receptors is known to activate *I*_Ca,L_ [58].

The incubation of hBM-MSCs with IL-1β displayed a tendency to increase the inward nifedipine-sensitive current; however, this increase was not statistically significant.

The inhibition of L-VOCCs with nifedipine was reported to downregulate the proliferation of chondrocytes [38], suggesting the functional involvement of these channels. However, in our study, L-VOCCs activity was not detected in chondrocytes incubated in proliferation media. Notably, we observed a significant increase in outward currents when chondrocytes were incubated in chondrogenic media. We suppose that components of chondrogenic media, such as dexamethasone [59,60], high glucose concentration [61], or streptomycin [62], could enhance potassium outward currents, which might explain our observations. Further, incubation of chondrocytes with TGF-β3 resulted in inward currents that were not sensitive to nifedipine. One possible reason for this could be the downregulation in potassium channel expression by TGF-β [63]. The total membrane current is a sum of inward and outward currents. Therefore, the downregulation of potassium outward currents will result in the total inward membrane current.

Our data showed that neither the application of proliferation nor chondrogenic media, TGF-β3 nor IL-1β stimulation, have changed L-VOCCs activity in chondrocytes.

We explored how TGF-β3-mediated increase in iCa^2+^ can modulate the gene expression of the CaV1.2 (*CACNA1C* gene) and the SERCA pump (*ATP2A2* gene), which regulate Ca^2+^ influx and balance it. TGF-β3 led to a significant increase in *ATP2A2* gene expression in both hBM-MSCs and chondrocytes. It is important to note that *ATP2A2* gene expression does not necessarily correlate with SERCA2 protein levels or the functional activity of this pump.

In chondrocytes exposed to TGF-β3 for 24 h, we found the downregulation of *CACNA1C* gene expression, whereas no change was observed in hBM-MSCs. Recent publications showed that treatment with TGF-β for 21 days resulted in a significant increase in *CACNA1C* gene expression in hBM-MSCs and OA chondrocytes [32]. Even 2 ng/mL of TGF-β for 96 h increases both gene and protein expression of L-VOCCs subunit CaV1.2 in human adipose-derived MSCs [64]. These data suggest that the effects of TGF-β3 on *CACNA1C* gene expression might depend on the duration of exposure to TGF-β3.

Since IL-1β regulates the gene expression of TRPA1 in rat chondrocytes, human intervertebral disc tissue and epithelial sodium channels in rat alveolar epithelial cells [29,65,66], we explored how it regulates the gene expression of *CACNA1C* and *ATP2A2* in hBM-MSCs and chondrocytes. We observed an increase in the *ATP2A2* gene expression in hBM-MSCs when exposed to IL-1β. In our study, a 24 h incubation with 10 ng/mL IL-1β resulted in a decrease in *CACNA1C* gene expression in chondrocytes. In contrast, previous research reported that a 48 h treatment with IL-1β at a concentration 10 times lower (1 ng/mL) than that used in our study led to an increase in *CACNA1C* gene expression in bovine chondrocytes [67]. The differing results regarding the effects of IL-1β on *CACNA1C* may be attributed to different concentrations and exposure durations. These differences highlight the need for additional studies to understand how IL-1β influences *CACNA1C* expression under various exposure conditions.

Both hBM-MSC and chondrocytes were obtained from tissues removed during articular surgery in OA patients. The limited availability of “healthy” cells remains a well-recognized challenge [68]. It is known that OA cartilage-derived chondrocytes exhibit altered intracellular signaling [35,69], reduced regenerative potential [35,70,71] and accelerated cellular senescence [71]. However, OA patient-derived autologous cells are commonly used in cell therapy approaches [72,73]. Moreover, the focus of the present study represents remodeling mechanisms, rather than a comparison between healthy and disease states.

**Figure 5:**
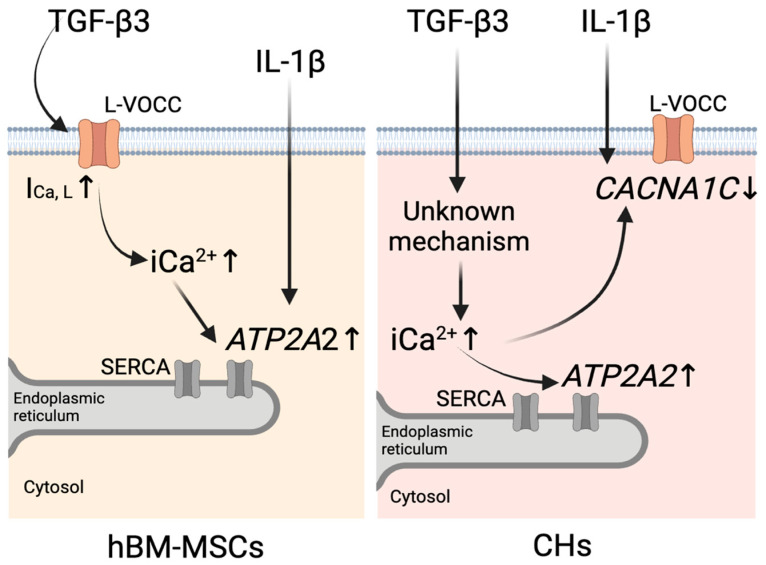
Schematic representation of the proposed mechanisms of calcium homeostasis regulation by TGF-β3 and IL-1β in hBM-MSCs (**left**) and chondrocytes (CHs) (**right**). In hBM-MSCs, TGF-β3 increases intracellular calcium (iCa^2+^) levels by enhancing L-type calcium current (*I*_Ca,L_), which leads to *ATP2A2* upregulation. IL-1β upregulates *ATP2A2* expression. In CHs, TGF-β3 increases iCa^2+^ via an unknown mechanism, which leads to upregulation of *ATP2A2* and downregulation of *CACNA1C*. IL-1β downregulates *CACNA1C* expression. The image was created with BioRender.com (https://www.biorender.com/, accessed on 26 February 2025).

In summary, our findings in hBM-MSCs indicate that TGF-β3 exposure elevates iCa^2+^, at least partially, due to enhanced *I*_Ca,L_ (Figure 5, left). While the expression of *CACNA1C*, a subunit of L-VOCCs, remains unchanged, we suggest that TGF-β3 exposure increases the functional activity of the L-VOCCs already present. Moreover, TGF-β3 results in the upregulation of *ATP2A2* expression (Figure 5, left). Upregulation of *ATP2A2* may provide the initial step in a cellular response for the reduction in elevated iCa^2+^. In chondrocytes, TGF-β3 exposure also elevates iCa^2+^ but without changes in *I*_Ca,L_ (Figure 5, right), indicating different pathways for iCa^2+^ elevation than in hBM-MSCs. Moreover, TGF-β3 downregulates *CACNA1C* expression while upregulating *ATP2A2* (Figure 5, right). This suggests involvement of two mechanisms of elevated iCa^2+^ reduction in chondrocytes: by enhancing Ca^2+^ reuptake from the cytosol into the endoplasmic reticulum through *ATP2A2* upregulation and by downregulation of *CACNA1C*.

IL-1β did not change the levels of iCa^2+^ or *I*_Ca,L_ in either hBM-MSCs or chondrocytes. However, the upregulation of *ATP2A2* gene expression observed in hBM-MSCs (Figure 5 left) and the downregulation of the *CACNA1C* gene in chondrocytes (Figure 5 right), as responses to stimulation with IL-1β, suggest that its mechanism of action is also associated with alterations in Ca^2+^ signaling, which different types of cells handle in different ways.

It is interesting to note that there is some degree of convergence of TGF-β3 and IL-1β action on calcium homeostasis: in hBM-MSCs both cytokines increase the expression of the *ATP2A2* gene, while in chondrocytes, both increase the expression of the *CACNA1C* gene.

We conclude that both TGF-β3 and IL-1β influence iCa^2+^ homeostasis in hBM-MSCs and chondrocytes during chondrogenesis in a cell-type-dependent manner.

## Data Availability

The data supporting intracellular calcium levels and gene expression study findings can be found at State Research Institute Centre for Innovative Medicine, Department of Regenerative Medicine. The data supporting electrophysiological study findings can be found at Vilnius University, Institute of Biosciences.
